# Dynamic response of RNA editing to temperature in grape by RNA deep sequencing

**DOI:** 10.1007/s10142-019-00727-7

**Published:** 2019-11-19

**Authors:** Aidi Zhang, Xiaohan Jiang, Fuping Zhang, Tengfei Wang, Xiujun Zhang

**Affiliations:** 1 Key Laboratory of Plant Germplasm Enhancement and Specialty Agriculture, Wuhan Botanical Garden of the Chinese Academy of Sciences, Wuhan, 430072 China; 2 University of Chinese Academy of Sciences, Beijing, 100049 China

**Keywords:** RNA editing, Stress, Temperature, grape, RNA sequencing, PPR

## Abstract

RNA editing is a post-transcriptional process of modifying genetic information on RNA molecules, which provides cells an additional level of gene expression regulation. Unlike mammals, in land plants, RNA editing converts C-to-U residues in organelles. However, its potential roles in response to different stressors (heat, salt, and so on) remains unclear. Grape is one of the most popular and economically important fruits in the world, and its production, like other crops, must deal with abiotic and biotic stresses, which cause reductions in yield and fruit quality. In our study, we tested the influence of the environmental factor temperature on RNA editing process in the whole mRNA from grape organelle. In total, we identified 122 and 627 RNA editing sites in chloroplast and mitochondria respectively with the average editing efficiency nearly ~ 60%. The analyses revealed that number of non-synonymous editing sites were higher than that of synonymous editing sites, and the amino acid substitution type tends to be hydrophobic. Additionally, the overall editing level decreased with the temperature rises, especially for several gene transcripts in chloroplast and mitochondria (*matK*, *ndhB*, etc.). We also found that the expression level of most PPR genes decreased with the temperature rises, which may contribute to the decline of RNA editing efficiency at high temperature. Our findings suggested that the RNA editing events were very sensitive to heat stress; the changes of amino acid in RNA editing genes may contribute to the stress adaption for grape.

**Electronic supplementary material:**

The online version of this article (10.1007/s10142-019-00727-7) contains supplementary material, which is available to authorized users.

## Introduction

RNA editing is a post-transcriptional process that could alter the nucleotide sequence of gene transcripts, potentially diversifies the transcriptome and proteomes beyond the genomic blueprint (Takenaka et al. 2013b). In mammals, RNA editing occurs in nucleus transcripts; adenosine-to-inosine (A-to-I) editing is the most abundant type of RNA editing catalyzed by the protein family called adenosine deaminases acting on RNA (ADAR). Mutants lacking the ADAR enzyme exhibited behavior alterations including defects in flight, motor control, and mating (Palladino et al. 2000). However, in plant, RNA editing primarily occurs in organelle transcripts, and the RNA editing type is dominated by cytosine-to-uracil (C-to-U) RNA editing, in ferns and mosses; it also changes U nucleotides to C nucleotides. RNA editing was first documented over a decade ago in mitochondria as sequence differences between DNA and RNA (Covello and Gray 1989; Gualberto et al. 1989; Hiesel et al. 1989), and then, a number of editing sites in both two organelles (plastids and mitochondria) were subsequently reported in all land plants, including all major plant lineages from the bryophytes to gymnosperms and in all angiosperms.

RNA editing has various biological functions; recent studies reported that RNA editing plays important roles in various plant developmental processes and evolutionary adaptation, including organelle biogenesis, signal transduction, and adaptation to environmental changes (Fujii and Small 2011; Hammani and Giege 2014). Accordingly, numerous evidences also reported that RNA editing on transcripts was responsive to various environmental stressors, such as temperature, salt, and so on; it is assumed that RNA editing may well affect the second structure of selected transcripts in response to various stresses (Garrett and Rosenthal 2012a, b; Karcher and Bock 2002; Kurihara-Yonemoto and Handa 2001; Kurihara-Yonemoto and Kubo 2010; Rieder et al. 2015; Riemondy et al. 2018; Rodrigues et al. 2017). It was also demonstrated that mutants with impaired editing of specific sites exhibited strong deleterious phenotypes, even lethality (Tang et al. 2017). Since the advent of next-generation sequencing technologies, RNA-seq data becomes a comprehensive, precise, and low-cost approach for transcriptome profiling and variant analysis; tens of thousands of editing sites have been identified in more and more plants (Edera et al. 2018; Grimes et al. 2014; Picardi et al. 2010). The growing of public RNA-seq data also provides an excellent opportunity to investigate the effect of RNA editing on organelle function and evolution (Edera et al. 2018; Smith 2013); a comprehensive picture of C-to-U RNA editing sites was described in angiosperm mitochondria, revealing that RNA editing sites are conserved across angiosperms but some species-specific sites still exist.

RNA editing in plant organelles involves mainly the deamination of C-to-U by specific pentatricopeptide repeat (PPR) proteins with several additional non-PPR protein factors that are both encoded in the nuclear genome (Ichinose and Sugita 2017; Yan et al. 2018). PPR proteins have been reported to contain conserved domains to edit mRNA of organelle genes and formed an extended family (over 400 members) during plant evolution (Manna 2015). Studies have demonstrated that 30–40 amino acid repeated motifs in PPR proteins are responsible for the specificity of the editing reaction, whereas the C-terminal E domain is required for the editing reaction to occur (Ichinose and Sugita 2017). PPR proteins are classified into different subclasses based on their domain architecture, which is often a reflection of their function. Several members of the PPR protein family have been investigated; MEF9 (mitochondrial editing factor 9) is required for RNA editing of mitochondrial mRNA at sites of nad7-200 (Sugita et al. 2013; Takenaka 2010); CLB19 (chloroplast biogenesis 19) is needed for editing of specific sites in plastid clpP (chloroplast protease) and rpoA (chloroplast RNA polymerase) transcripts respectively; the PPR protein MEF32 (mitochondrial editing factor 32) binds to a specific RNA sequence motif of their target editing sites to improve the efficiency of RNA editing (Takenaka et al. 2013a). It remains unclear whether the large number of similar PPR proteins predicted to be targeted to mitochondria also specify editing sites (Schmitz-Linneweber and Small 2008). Editing specificity factors are probably the best subjects for understanding the basis for PPR recognition of specific RNA sequences because the target site is precisely defined. Additionally, many PPR mutants can further alter their morphological appearances under stress conditions compared with the wild type (Tan et al. 2014; Tang et al. 2017).

Grape (*Vitis vinifera* L.) is one of the most popular and economically important fruits in the world. Grape production, like other crops, however, must deal with various abiotic and biotic stresses, which cause its reductions in yield and fruit quality. It remains unclear that whether the RNA editing events could response to various stresses, such as temperature and water. In this study, we tested the influence of the environmental factor temperature on RNA processing based on whole mRNA deep-sequencing data. With the temperature increasing, reduced RNA editing efficiency was detected significantly, which may resulted from temperature-sensitive expression or stability of the RNA editing factors. Environmental cues, in this case temperature, rapidly reprogram the grape transcriptome through RNA editing, presumably resulting in altered proteomic ratios of edited and unedited proteins. Our findings also suggest that the changes of amino acid in these genes may contribute to the stress adaption for grape.

## Material and methods

### Data collection

All data sets used in this study are publicly available. The RNA-seq data of grape leaves that treated with different temperatures (25 °C, 35 °C, 40 °C, and 45 °C) were downloaded from https://www.ncbi.nlm.nih.gov/bioproject/PRJNA350310. The genome sequences of grape mitochondria and chloroplast and corresponding genome annotation files in “tbl” format were downloaded from the NCBI data repository (https://www.ncbi.nlm.nih.gov; accession numbers: NC_012119.1, NC_007957.1). The whole genome and annotation file of grape were also downloaded from the NCBI data repository for expression analysis (Jaillon et al. 2007).

### Pre-analysis of transcriptome data

We performed the RNA editing sites identification for grape mitochondria and chloroplast separately, and the identification process was split into two steps. For each organelle genome, firstly, we aligned the transcriptome data against the reference and called SNPs; secondly, the RNA editing sites were identified based on the called SNPs-calling results. In order to increase the sequencing depth, we merged the three duplicates under one condition into one sample. Take mitochondria as an example, the quality control of paired-end Illumina sequencing data were firstly evaluated by NGSQCToolkit and low-quality sequence data were filtered out (cutOffQualScore<20) (Patel and Jain 2012), and the treated cleaned reads were aligned to the reference mitochondria genome using the HISAT2 software (default parameters) (Kim et al. 2015). Samtools (Li et al. 2009) was used to index, merge, sort, remove, format convert, mpileup, and remove duplications against the aligned data. Afterwards, the bcftools was used to perform SNP-calling based on the treated bam files, and the VCF files were generated (Narasimhan et al. 2016) for subsequent analysis.

### Identification of RNA editing sites

Based on the SNP-calling results (in “VCF” format) and genome annotation files (in “tbl” format), we utilized the REDO tool to identify the RNA editing sites under default parameter values (Wu et al. 2018). REDO is a comprehensive application tool for identifying RNA editing events in plant organelles based on variant call format files from RNA-sequencing data. REDO only uses the variant call format (VCF) files (records for all sites), the genome sequence file (FASTA format), and the gene annotation file (feature table file in “tbl” format, www.ncbi.nlm.nih.gov/projects/Sequin/table.html) as inputs. Afterwards, the raw variants are filtered by ten rule-dependent filters and statistical filters to reduce the false positives as the following steps: (1) quality control filter, (2) depth filter (DP > 4), (3) alt proportion filter (alt proportion < 0.1), (4) multiple alt filter, (5) distance filter, (6) spliced junction filter, (7) indel filter, likelihood ratio (LLR) test filter (LLR <10), (8) Fisher’s exact test filter (*p* value < 0.01), and (9) complicated filter model. Finally, all filtered RNA editing sites were identified and their corresponding annotation information files were also generated at the same time.

### Characteristic statistics and identified RNA editing sites

For each organelle, the resulted editing sites were used for further statistics and feature analysis, including statistics of editing number, editing type, codon position, amino acid changes, and involved genes. The value of RNA editing efficiency at one site was expressed as the proportion between edited transcripts and total transcripts. If one site was edited, the C/G base (wild type) should be altered to the T/A base (edited type), since one editing site could be detected hundreds of times via sequencing, the number of wild type (C/G) or edited type (T/A) of bases could then be counted at this particular site, then the editing efficiency at one site could then be calculated by the formula: depth of edited bases (T and A)/total read depth of bases. Furthermore, we compared the RNA editing efficiency between each two conditions and identified the editing sites with statistical significance (*p* value < 0.01). In order to decipher the tendency of RNA editing efficiency for each organelle, cluster analysis and heatmap plotting were also provided based on the RNA editing efficiency matrix. Values of editing efficiency matrix were normalized by subtracting the row-wise mean from the values in each row of data and multiplying all values in each row of data by standard deviation value. A heatmap was plotted in all samples using “pheatmap” function in R, the distance matrix of different samples was calculated using “dist” function with the default Euclidean method, and the hierarchical clustering was computed using “hclust” function. Genes with significantly changes in editing sites were picked out for further functional analysis.

### Expression analyses of PPR genes

Transcriptome analysis of RNA-seq data used in our study was also performed for measuring and comparing the levels of gene expression of PPR genes. One PPR protein sequence (UniProtKB ID: Q9SAD9) of *Arabidopsis thaliana* was used as query to search against the *Vitis vinifera* protein databases using BLASTp with default settings. All positive hits were retrieved for gene function annotation to blast against the Swiss-Prot protein database. The protocol that described in previous study (Pertea et al. 2016) was used for transcriptome analysis. Concretely, treated reads from each sample were mapped to the reference genome with HISAT2; Stringtie was used for transcript assembly; Samtools was used to index, merge, sort, remove, format convert, mpileup, and remove duplications against the aligned data with default parameters; and finally, Ballgown was used to determine deferentially expressed genes between each two conditions. Gene expression levels are measured by FPKM (fragments per kilobase of transcript per million mapped reads), expression values of PPR proteins were also normalized by the method mentioned above, and a heatmap was plotted in all samples using “pheatmap”function in R.

### Statistical analysis

Two-tailed Wilcoxon rank-sum test was used to perform the pairwise comparison of RNA editing efficiency between each two neighboring conditions. As for the pairwise comparison for each editing site, Fisher’s exact tests were used.

## Results

### Alignment of transcriptome data

**Fig. 1:**
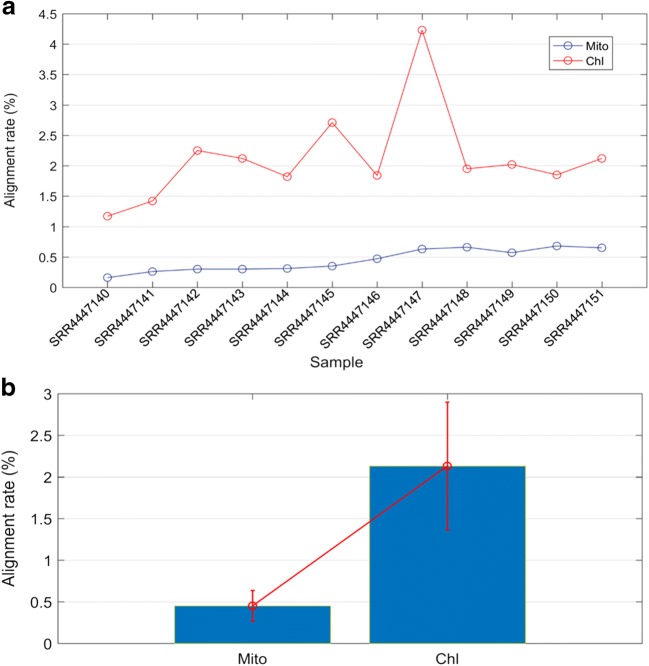
The mapping rates of transcriptome data to mitochondrion and chloroplast genomes. **a** The statics of reference-guided mapping rate. The *y*-axis represents mapping rate of each sample, and the *x*-axis represents each sample. **b** The bar figure of the statics of reference-guided mapping rate. The *y*-axis represents mapping rate of each sample, and the *x*-axis represents organelle (Chl: chloroplast; Mito: mitochondria)

There were a total of 12 RNA-seq samples that treated with different temperatures (25 °C, 35 °C, 40 °C, and 45 °C) in our study; each condition has three replicates. We aligned the transcriptome data to the organelle reference genome respectively. The size of mitochondrion genome is about 773,279 bp, encoding 158 genes, for each sample; there were about 80,000 reads mapped to reference with mapping rate about 0.45% (std = 0.185). For chloroplast, the size of its genome is about 160,928 bp, encoding 120 genes. There were about 400,000 reads mapped to reference with mapping rate about 2.13% (std = 0.7681). Generally speaking, more genes are located in mitochondrion compared with chloroplast, and then, the mapping reads of mitochondrion should be more than that of chloroplast; however, it turned out, an opposite result beyond our expectation, chloroplast has a higher mapping rate significantly, which may be due to sources of samples; more chloroplast mRNA in leaves were extracted and sequenced in this study. The statics of reference-guided mapping rate was shown in Fig. 1.

### Identification of RNA editing sites

In order to increase the sequencing depth and reliability of editing sites, the resulting bam files of three replicates under one condition were merged for subsequent identification of RNA editing sites. Based on the SNP-calling results and organelle genome annotation file, a total of 749 RNA editing sites were identified in both organelles; however, a few editing sites only appeared under certain condition. Take samples at 25 °C temperatures in chloroplast as illustration, the attributes of RNA editing sites were shown in Fig. S1. For mitochondrion, there were 627 RNA editing sites identified, involving 53 genes; the number of RNA editing sites identified at different temperatures (25 °C, 35 °C, 40 °C, and 45 °C) correspond to 468, 509, 563, and 582, along with the increment of temperature, the number of sites increased obviously, as shown in Table 1. In contrast, there were only 122 editing sites identified in chloroplast, involving 43 genes; the number of sites did not appear to be rising along with temperature increment; 95 editing sites were identified under three conditions (25 °C, 35 °C, 40 °C); and only 82 sites were identified under 45 °C temperature. The statistics results showed that most of editing sites were C-to-U; for chloroplast, 97 out of 122 editing sites were C-to-U, the second-most type was G-to-A (5 out of 122); for mitochondrion, 602 out of 627 editing sites were C-to-U, the second-most type was G-to-A (25 out of 627). The detailed information of RNA editing sites in all chloroplast and mitochondrion samples were listed in Table S1 and Table S4. There were two possibilities for higher number of editing sites in mitochondrion; one is data bias in sequencing depth; the mapping rate under higher temperature is higher than that of lower temperature, which may give rise to generation of new editing sites; another is upregulation of several PPR proteins; detailed information can be found in the sixth part of results.

**Table 1:** The statistics of identified RNA editing sites in mitochondrion and chloroplast

Organelle	Samples	Total	Phase(1,2,3)	Silent	Non-silent
Chl	T25	95	14,77,12	6	89
	T35	95	11,76,16	9	86
	T40	95	15,74,36	10	85
	T45	82	14,66,31	8	74
Mito	T25	468	137,291,62	49	419
	T35	509	156,305,73	59	450
	T40	563	170,342,84	63	500
	T45	582	175,355,84	61	521

### Characteristics of the statistics for RNA editing sites

**Fig. 2:**
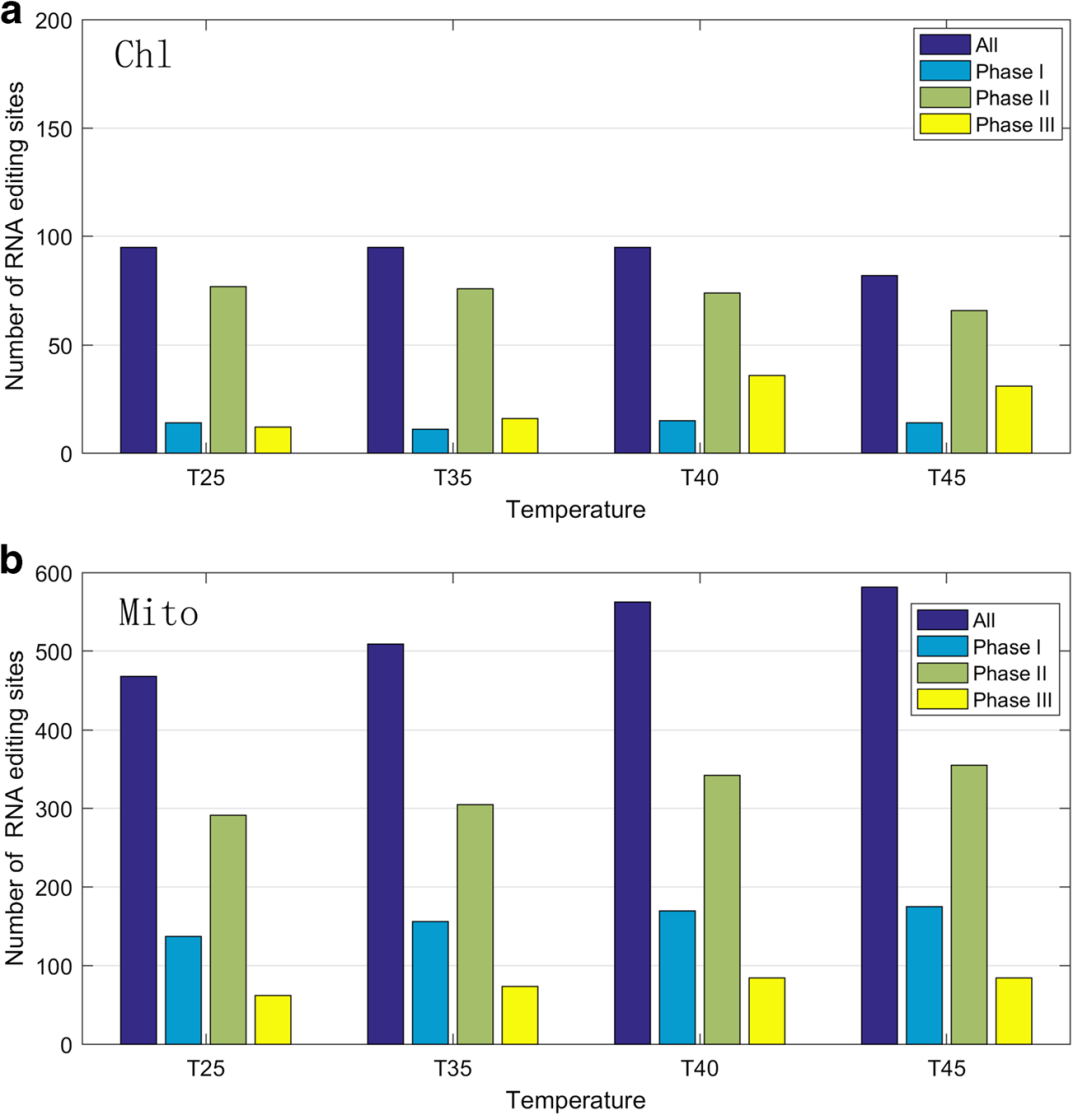
Codon position statistics of RNA editing sites were shown in **a** (Chl: chloroplast) and **b** (Mito: mitochondria) respectively

We also found that RNA editing occurred in second codon position was mainly the largest in both organelles, followed by first codon position except three conditions (35 °C, 40 °C, and 45 °C) of chloroplast, as shown in Fig. 2. In mitochondrion, globally, 30%, 58%, and 12% of the 627 identified editing sites were found at first, second, and third codon positions, respectively. Similarly, in chloroplast, 14%, 76%, and 10% of the 122 identified editing sites were found at first, second, and third codon positions, respectively. Furthermore, the statistics of editing type showed that the majority (~ 95%) of the editing events resulted in non-synonymous codon changes. Interestingly, we found that the amino acid changes tend to be hydrophobic; the change from hydrophilic to hydrophobic was the highest, followed by the change from hydrophobic to hydrophobic; take condition of T25 for an example, the proportion of hydrophobic2hydrophobic: hydrophilic2hydrophilic: hydrophobic2hydrophilic: hydrophilic2hydrophobic was 114:49:36:206 in mitochondrion, and 13:9:2:62 in chloroplast. In addition, about ~ 55% of the amino acid changes were hydrophilic2hydrophobic produced by editing sites mainly at second codon positions. The most amino acid changes were Ser-to-Leu and Pro-to-Leu; serine is hydrophilic, whereas Leucine and Proline are both hydrophobic. The above results were in good agreement with previous studies (Takenaka et al. 2013b; Yan et al. 2018), which demonstrated that the RNA editing caused an overall increase in hydrophobicity of the resulting proteins.

### Reduced RNA editing efficiency with the temperature rises

**Fig. 3:**
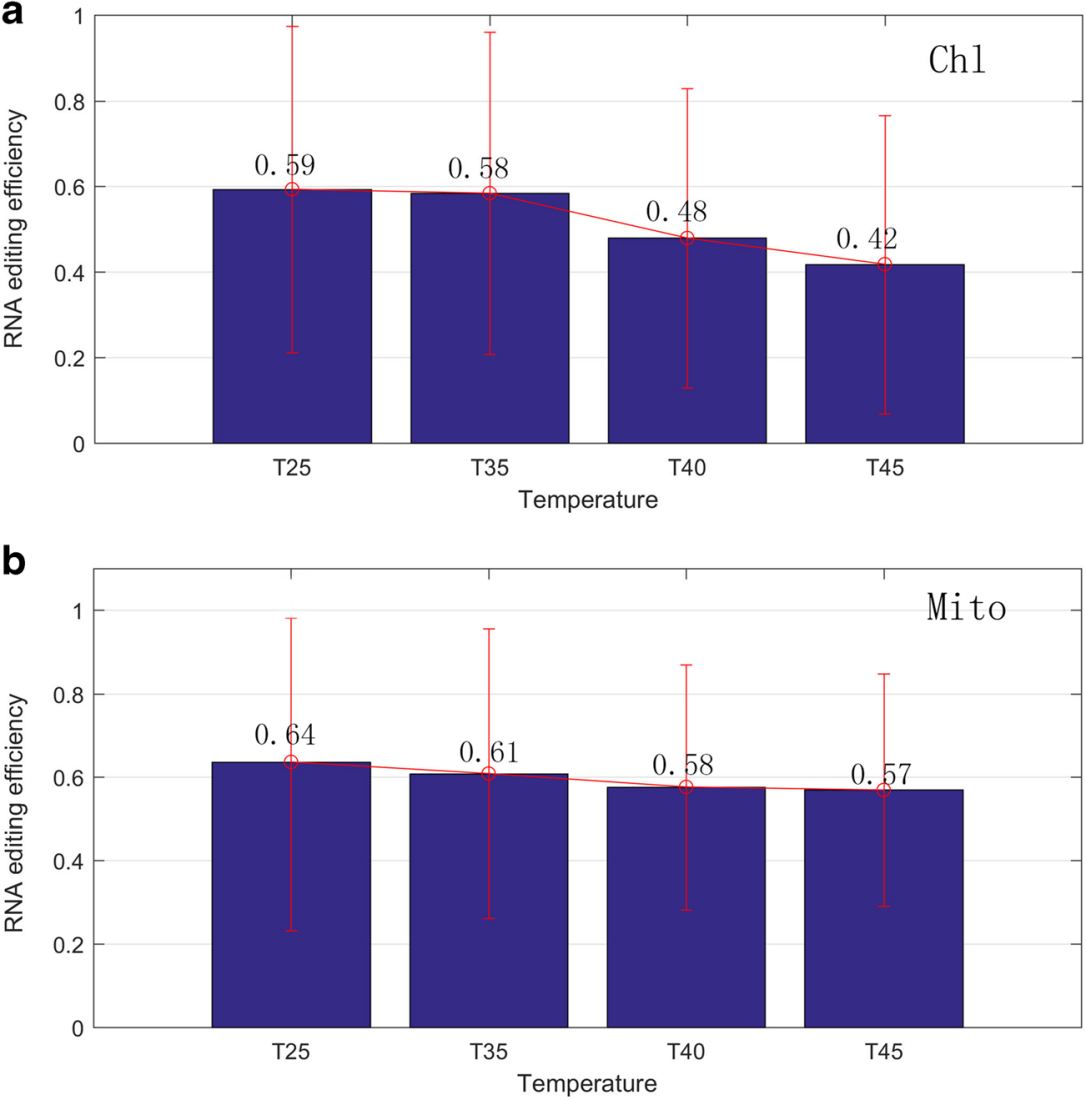
Average editing efficiency of all the RNA sites under four temperature conditions (25 °C, 35 °C, 40 °C, and 45 °C) were shown in **a** (Chl: chloroplast) and **b** (Mito: mitochondria) respectively

**Fig. 4:**
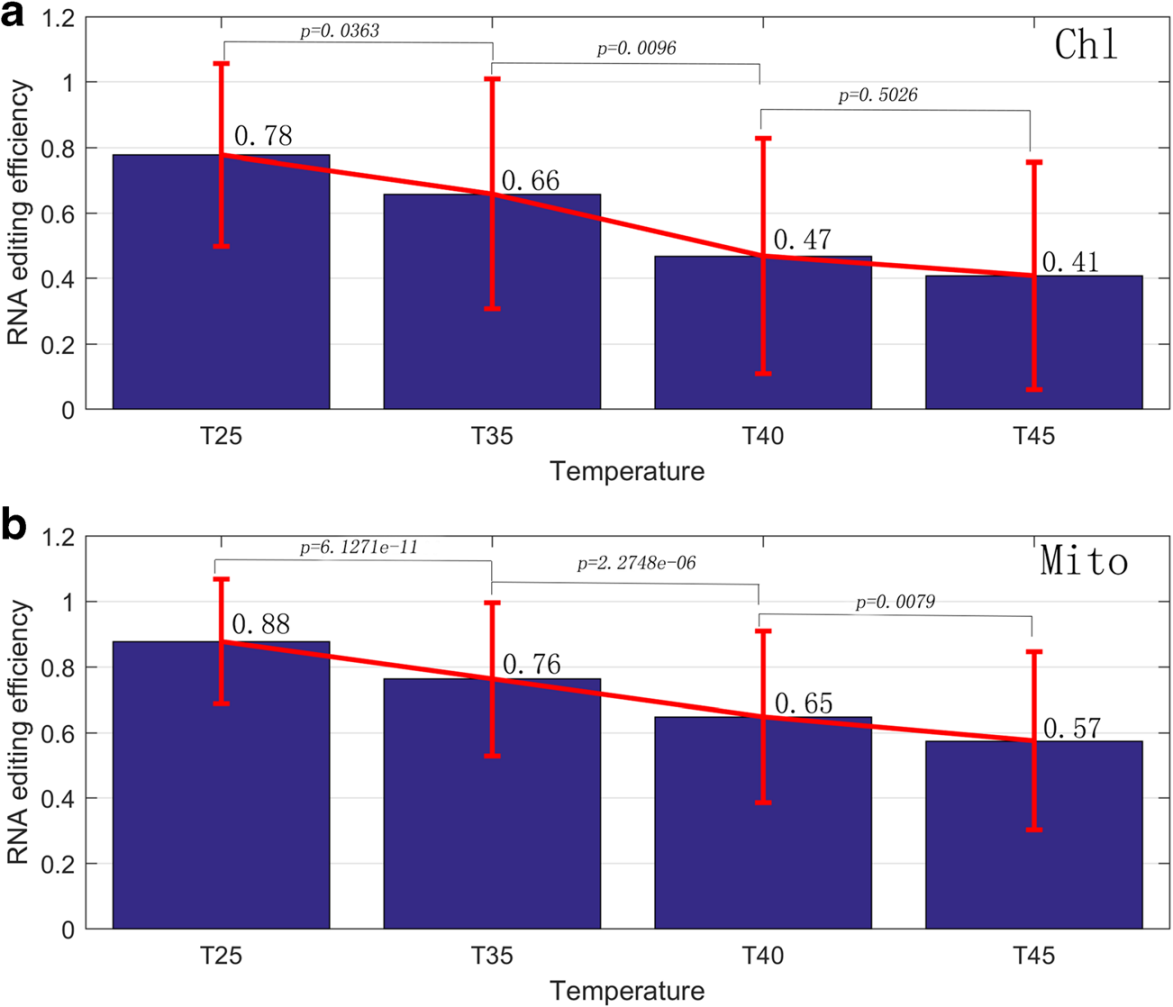
RNA editing efficiency pattern of 175 “step-down” editing sites. **a** RNA editing efficiency of 30 “step-down” editing sites in chloroplast. **b** RNA editing efficiency of 145 “step-down” editing sites in mitochondria. Two-tailed Wilcoxon rank-sum test was used to perform the pairwise comparison

**Fig. 5:**
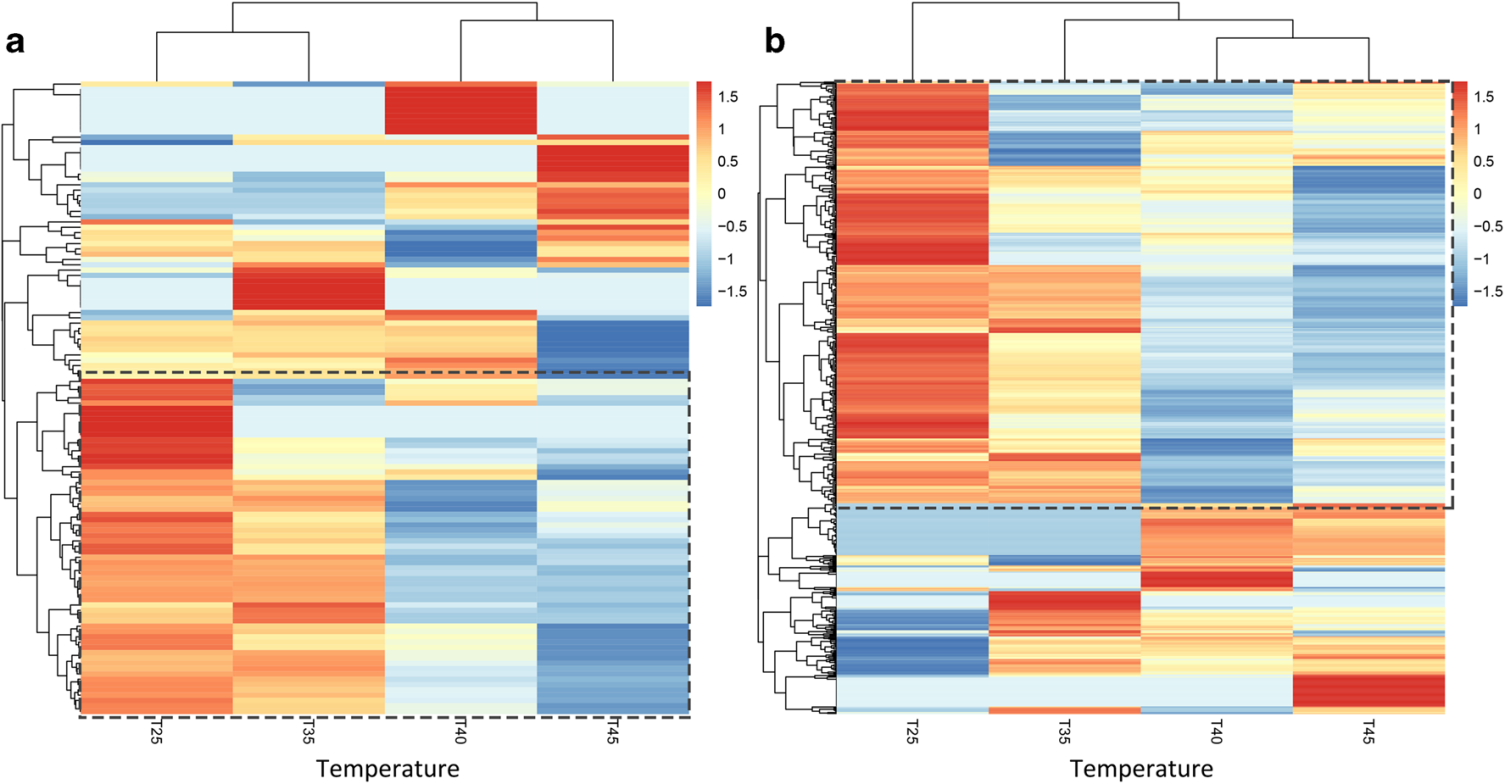
Heatmap of RNA editing efficiency under four temperature conditions (25 °C, 35 °C, 40 °C, and 45 °C) were shown in **a** (Chl: chloroplast) and **b** (Mito: mitochondria) respectively. The *x*-axis represents different samples, and the *y*-axis represents editing sites. Editing sites with reduced efficiency are indicated by black dotted box, as shown in Fig. 6 and Fig S1

We also performed statistics and cluster analysis for the RNA editing efficiency. On the whole, the average efficiency of RNA editing sites was about 0.56. For chloroplast and mitochondrion, the average RNA editing efficiency was 0.59, 0.58, 0.48, and 0.42 and 0.64, 0.61, 0.58, and 0.57 respectively under four conditions (25 °C, 35 °C, 40 °C, and 45 °C). With the increase of temperature, the average editing efficiency both declined gradually, as shown in Fig. 3. Actually, since only a large part of editing site demonstrated strongly decreased RNA editing efficiency, the rest editing sites, however, have no significant changes, then no significant differences was detected on the whole level for both organelles. In addition, we separated out the RNA editing sites with “step-up” and “step-down” editing efficiency, where “step-up” denotes the editing efficiency increases as the temperature increases; conversely, “step-down” denotes the editing efficiency decreases as the temperature increases. Finally, a total of 244 sites editing sites were identified in both organelles, and most of these sites have “step-down” editing efficiency. There were 175 sites demonstrated the trend of decreasing (30 for chloroplast, 145 for mitochondrion), whereas 69 sites demonstrated the trend of increasing (11 for chloroplast, 58 for mitochondrion). For the “step-down” editing sites, two-tailed Wilcoxon rank-sum test was used to perform pairwise comparisons, a remarkable significance (*p* < 0.05) was detected in the comparison of RNA editing efficiency between each two neighboring conditions except for T40-T45 of chloroplast, as shown in Fig. 4. Furthermore, the cluster analysis results also showed that the clustering relationship among samples agreed with the changes of temperatures, and there was a large area demonstrated the trend of decreasing, as shown in Fig. 5. In total, our results suggested that RNA editing process was acutely sensitive to temperature; it is possible that differential RNA editing is one process that allows plants such as grape to rapidly adapt to varying environmental temperatures. Detailed information of RNA editing efficiency was listed in Table S2 and S5. Pairwise comparison of editing allele proportion in all samples was listed in Table S3 and S6.

### Genes with changes of RNA editing efficiency

**Fig. 6:**
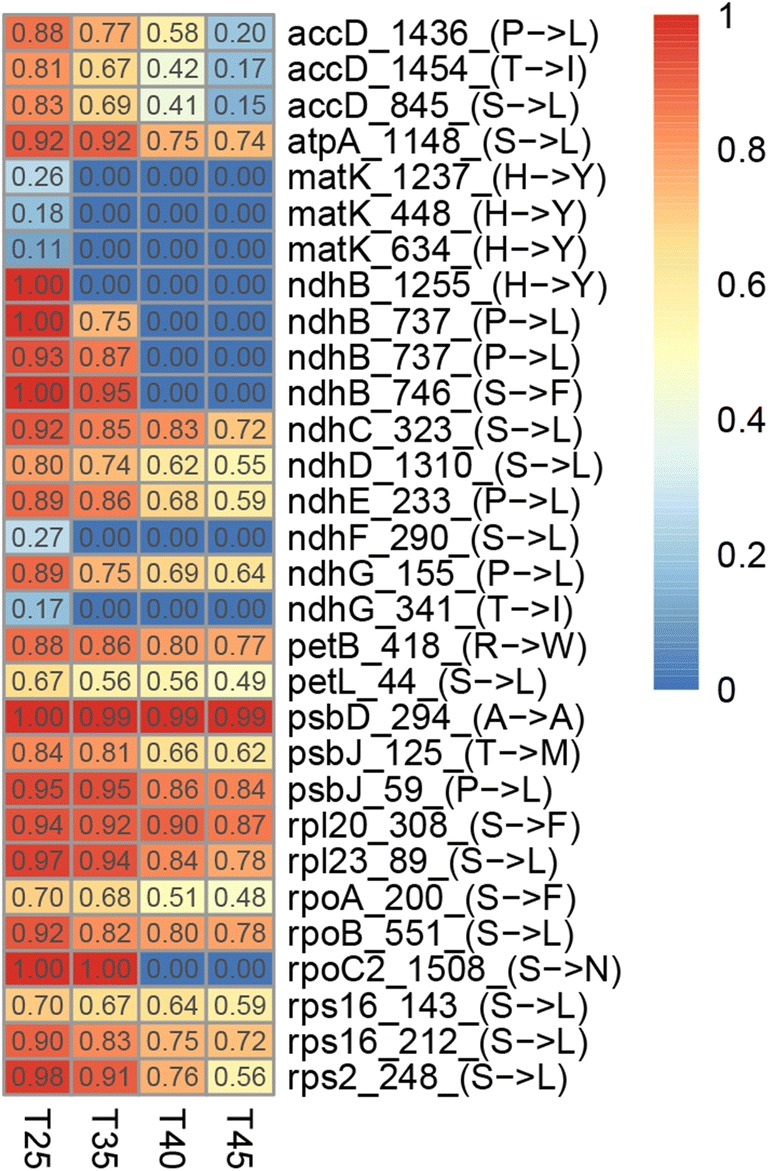
Reduced efficiency patterns of RNA editing in chloroplast. The number of RNA editing efficiency is indicated inside the box; the name of editing sites is concatenated by gene symbol, site of position, and type of amino acid change

We annotated the involved genes with editing sites of “step-up” and “step-down” RNA editing efficiency. Hence, a total of 68 genes were annotated (25 for chloroplast, 43 for mitochondrion), as shown in Fig. 6 and Fig. S2. For chloroplast, several genes have more editing sites, especially *maturase K* (*matK*) and *NADH dehydrogenase subunit 2* (*ndhB*) genes; both genes have four changed editing sites. All the sites of *ndhB* gene (Chl-100212, Chl-148651, Chl-101400, Chl-101409) were C-to-U editing type and demonstrated the trend of decreasing; the corresponding amino acid changes were His-to-Tyr, Pro-to-Leu, Ser-to-Phe, and Pro-to-Leu, the four amino acids all changed to be hydrophobic. Whereas three sites of *matK* gene demonstrated the trend of decreasing, one site showed a rising trend. For mitochondrion, more genes have editing efficiency changed sites, such as *NADH dehydrogenase* gene family (*nad4/5/7*), *ATPase* gene family (*atp6/9*), *heme trafficking system membrane* gene family (*ccmB/C/FC/FC/FN*), *mitochondrial Cytochrome c oxidase* gene family (*cox1/2/3*), and *ribosomal* gene family (*rps4/7*).

*ndhB* gene encodes components of the thylakoid *ndh* complex which purportedly acts as an electron feeding valve to adjust the redox level of the cyclic photosynthetic electron transporters. *ndhB* gene contains by far the higher number of editing sites, 10 in *Arabidopsis*, probably because the proofreading mechanism that ensures identical sequences of the two inverted repeated regions of plastid DNA makes improbable the fixation of C-to-U back mutations (Martin and Sabater 2010). Previous studies reported that *ndh* complex is related to stress resistance; transgenic tobaccos defective in the *ndhB* gene have impaired photosynthetic activity at actual but not at high atmospheric concentrations of CO_2_ (Horvath et al. 2000). Furthermore, positive selection in *ndhB* gene was detected in ferns and angiosperms; the adaptive evolution may affect the energy transformation and light resistant; notably, many *ndh* genes were lost or pseudogenes in gymnosperm.

*matK* gene, single copy with the length of 1500 bp, usually encodes in the *trnK tRNA* gene intron, probably assists in splicing its own and other chloroplast group II introns (Hao da et al. 2010), involving genes include the transcripts of *trnK*, *trnA*, *trnI*, *rps12*, *rpl2*, and *atpF*; tRNAs and proteins produced by these genes are essential for chloroplasts to function properly. Similarly, *matK* gene also suffers adaptive evolution in angiosperms, which means a lot for the transcription process of related genes (Hao da et al. 2010). Thus, the changes of amino acid sites resulting from evolution or RNA editing may fine-tunes maturase performance.

### Expression analysis of RNA editing genes and PPR genes

**Fig. 7:**
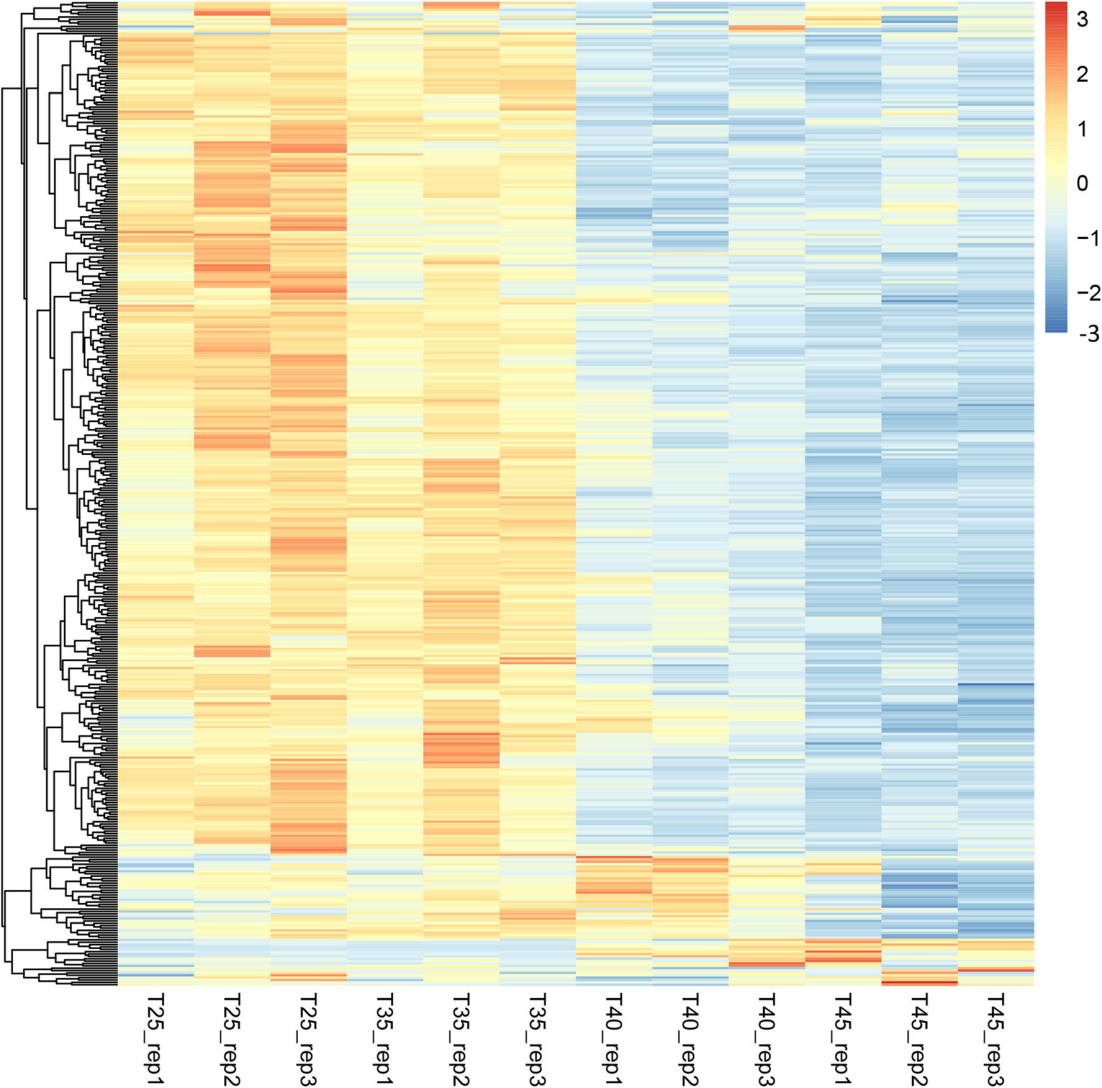
Heatmap of expression patterns of PPR proteins under four temperature conditions (25 °C, 35 °C, 40 °C, and 45 °C) with three replicates. The *x*-axis represents different samples, and the *y*-axis represents PPR proteins

Transcriptome analysis of RNA-seq data was also performed for measuring and comparing the levels of gene expression of RNA editing genes and PPR genes. However, for RNA editing genes, no expression difference was detected under different temperatures, suggesting that temperature stress only affect the RNA editing events and has no influence on expression level for those genes. In order to investigate the reason for the reduced RNA editing efficiency with the increasing of temperature, we also evaluated the expression of RNA editing genes and PPR proteins. After blast searching, a total of 419 proteins were identified as PPRs, and 414 PPR proteins were expressed. Interestingly, the expression level of most PPR proteins demonstrated a downregulated tendency along with the increasing of temperature, as shown in Fig. 7. Moreover, the PPR proteins expression pattern of conditions from 35 to 40 °C revealed a transition point of downregulation. Gene differential expression analysis between samples under two conditions (25 °C, 45 °C) was also performed, as shown in Table S7; a total of 31 PPRs were differently expressed (*p* value < 0.01, |FoldChange| > 2). Compared with 26 downregulated PPRs, there were still 5 upregulated PPR proteins, such as PPR proteins: GSVIVG01031345001 (PP284_ARATH), GSVIVG01012156001 (PP327_ARATH), GSVIVG01008664001 (PP425_ARATH), revealing different functions of PPR proteins. Hence, on a whole level, there is a positive correlation between the PPR proteins expression and RNA editing efficiency; it is reasoned that the reduced RNA editing efficiency may result from the dropped expression of most PPR proteins.

## Discussion

RNA editing is an important epigenetic mechanism by which genome-encoded transcripts are modified by substitutions, insertions, and/or deletions; it diversifies gnomically encoded information to expand the complexity of the transcriptome. It was first discovered in *kinetoplastid protozoa* followed by its reporting in a wide range of organisms (Maslov et al. 1994). RNA editing has various biological functions; it can promote RNA splicing by affecting the intron structures (Castandet et al. 2010; Farre et al. 2012). Some of those editing events regulate RNA degradation and microRNA (miRNA) function. In plant, RNA editing of gene transcripts also plays a central role during plant development and evolutionary adaptation. The alteration of editing at a specific site of a mitochondrial gene can harmfully impact plant growth, development, fertility, and seed development (Kim et al. 2009; Liu et al. 2013; Sun et al. 2015; Toda et al. 2012; Yap et al. 2015). Some evidences also suggested that environmental factors, e.g., rice to the cold and maize to the heat (Kurihara-Yonemoto and Kubo 2010; Nakajima and Mulligan 2001) affect RNA editing. In addition, RNA editing has its significance during evolution (Fujii and Small 2011) and has been suggested to play a role in plant adaptation to land conditions (e.g., extreme temperatures, UV, and oxidative stress) when plants colonized the land (Fujii and Small 2011; Hammani and Giege 2014).

With the advent of sequencing technology, RNA editing sites were identified in more and more organisms based on RNA deep sequencing, especially in plants. In our study, we characterized hundreds of RNA editing sites, and the statics of editing types indicated that RNA editing typically occurs as C-to-U conversion in translated regions of organelle (mitochondrial and chloroplast) mRNAs. Most of the C-to-U changes in the protein coding regions tend to locate at first, second positions, and the physicochemical property of amino acids was mostly modified. In addition, consistent with previous studies, we also found that amino acid changes tend to be hydrophobic; therefore, plant RNA editing is believed to act as an additional proofreading mechanism to generate fully functional proteins (Ichinose and Sugita 2017; Simpson and Maslov 1999; Takenaka et al. 2013b). In our study, the response of reduced RNA editing efficiency to high temperature also confirmed the relationship between environmental factors and RNA editing. It is reasonable that RNA editing may play roles in response to environmental stress through changing the corresponding gene functions. Our results also indicated that RNA editing was more prevalent at lower temperatures, which is also accord with a previous study in animal, that the phenotypic consequences of *ADAR* (RNA editing factors in animal) deficiency in *Drosophila melanogaster* indicated that RNA editing plays an integral role in temperature adaptation by sensing and acting globally on RNA secondary structure (Buchumenski et al. 2017). It is possible that differential RNA editing is one process that allows poikilothermic animals and higher plants, such as fly and grape, to rapidly adapt to varying environmental temperatures.

Natural DNAs are usually limited to double-stranded helical shapes, whereas RNA is different; the repertoire of possible RNA secondary and tertiary structures appears limitless. RNA secondary structure is strongly correlated with function. For an RNA molecule, its structure and corresponding thermodynamic stability both contribute to functional regulation (Bonetti and Carninci 2012). Dynamic RNA structures are acutely responsive and fundamentally sensitive to abiotic factors, such as temperature and metal ion concentration; hence, it is this mutability of RNA structure that allows RNA to act as a sensor and elicit rapid cellular responses (Wan et al. 2011). Our results suggest that RNA editing is acutely sensitive to temperature and that this response is partially affected by the thermo-sensitive secondary and tertiary RNA structures that direct editing. However, the molecular determinants underlying temperature-dependent RNA editing responses still need further study.

PPR proteins that encoded in the nuclear genome have been proven to play a central role in plant RNA editing (Hammani and Giege 2014). PPR proteins family is exclusively expanded in plants; over 450 members were detected in *Arabidopsis thaliana* and *Oryza sativa* (Lurin et al. 2004). Studies revealed that all the investigated PPR proteins are located in either plastids or mitochondria and specially bind to the cis element of the target RNA (Barkan and Small 2014). PPR mutants display various developmental defects (Saha et al. 2007). RNA editing specificity factors are probably the best subjects for understanding the basis for PPR recognition of specific RNA sequences because the target site is precisely defined (Manna 2015; Okuda and Shikanai 2012; Yagi et al. 2013a). Results in our study showed that the expression of most PPR proteins was dramatically decreased at elevated temperatures, partially, but not fully, explaining some RNA editing sites responses to temperature. Even so, there was still several PPR proteins demonstrated an upregulated tendency; these PPR proteins may play specific roles in the newly generated under higher temperature in mitochondria. All of the data on PPR editing factors have come from work on plastids, and it remains unclear whether the large number of similar PPR proteins predicted to be targeted also specify editing sites (Saha et al. 2007; Schmitz-Linneweber and Small 2008). Further effort needs to be put into clarify their involvement (or not) in RNA editing (Barkan and Small 2014; Cheng et al. 2016; Yagi et al. 2013b).

## Conclusion

C-to-U RNA editing is a highly conserved process that post-transcriptionally modifies mRNA, generating proteomic diversity. However, its potential role in response to different stressors (heat, salt, and so on) and growth development remains unclear. Our study suggested that RNA editing was responsive to environmental inputs in the form of temperature alterations. Using the angiosperms grape, we identified 122 and 627 RNA editing sites in chloroplast and mitochondria respectively with the average editing efficiency nearly ~ 60% and detected that acute temperature alterations within a normal physiological range result in substantial changes in RNA editing levels. Additionally, the analyses also revealed that number of non-synonymous editing sites were higher than that of synonymous editing sites, and the amino acid substitution type tends to be hydrophobic. The response of reduced RNA editing efficiency to temperature alterations further confirmed the relationship between environmental factors and RNA editing, which might be through intrinsic thermo-sensitivity of the RNA structures that direct editing or due to temperature-sensitive expression of the RNA editing enzyme (*PPR* genes). Environmental cues, in this case temperature, rapidly reprogram the grape organelles transcriptome through RNA editing, presumably resulting in altered structure or function of edited proteins. However, the underlying molecular mechanisms of stress adaptation for RNA editing still require further investigation.

## Electronic supplementary materials

Additional file 1: Table S1. Information of RNA editing sites in all chloroplast samples. (XLSX 36 kb)

Additional file 2: Table S2. A matrix for editing allele proportion of RNA editing sites in all chloroplast samples. (XLSX 16 kb)

Additional file 3: Table S3. Pairwise comparison of editing allele proportion in all chloroplast samples. (XLSX 76 kb)

Additional file 4: Table S4. Information of RNA editing sites in all mitochondria samples. (XLSX 140 kb)

Additional file 5: Table S5. A matrix for editing allele proportion of RNA editing sites in all mitochondria samples. (XLSX 40 kb)

Additional file 6: Table S6. Pairwise comparison of editing allele proportion in all mitochondria samples. (XLSX 359 kb)

Additional file 7: Table S7. Gene differential expression analysis between samples under two conditions (25°C, 45°C). (XLSX 12 kb)

Additional file 8: Fig. S1. The attributes of RNA editing sites in chloroplast illustrated by samples at 25°C temperatures. Fig. S2. Reduced efficiency patterns of RNA editing sites in mitochondria. (DOCX 852 kb)

## Abbreviations

A-to-I: Adenosine-to-inosine
C-to-U: Cytosine-to-uracil
*ADAR*: Adenosine deaminases acting on RNA
*PPR*: Pentatricopeptide repeat
*matK*: Maturase K
*ndhB*: NADH dehydrogenase subunit 2
*MORF*: Multiple organelle RNA editing factors
